# Mitochondrial mutation m.1555A>G and aminoglycoside-induced hearing loss in neonates: an integrative review

**DOI:** 10.3389/fped.2026.1973908

**Published:** 2026-10-28

**Authors:** P. E. Edison, M. J. Sankar, Michael Hall

**Affiliations:** 1 Department of Neonatal and Developmental Medicine, Singapore General Hospital, Singapore, Singapore; 2 Department of Pediatrics, All India Institute of Medical Sciences, ICMR National Institute of Child Health Research (ICMR-NICHR), New Delhi, India; 3 School of Health Sciences, University of Southampton, Southampton, United Kingdom

**Keywords:** aminoglycosides, genetic testing, hearing loss, sensorineural, m.1555A>G, neonatal screening, pharmacogenomic testing

## Abstract

**Objective:**

To evaluate the evidence related to the association between aminoglycoside-induced hearing loss and the mitochondrial mutation m.1555A>G, and to examine the potential clinical utility of pharmacogenetic prescription of aminoglycosides in neonates.

**Methods:**

An integrative literature review of m.1555A>G mutation and aminoglycoside-induced hearing loss in neonates, analysed using the Wilson-Jungner framework of population-based preventive screening.

**Results:**

The evidence base was observational and methodologically heterogeneous. Across population-based and epidemiological newborn cohorts, the study-level median frequency of m.1555A>G was 0.2% (range 0%–0.3%), with regional and ethnic variation. In a prospective multicentre study, aminoglycoside exposure was weakly associated with failed newborn hearing screen (NBHS) in carriers (OR: 1.26, 95% CI: 1.07–1.49, *p* = 0.0058). Pure tone hearing impairment was detected at 3 years in a very-low-birthweight carrier with prior exposure. Pilot data support both the analytical validity of prenatal screening using buccal swabs or blood samples and the feasibility of rapid NICU genotyping to inform aminoglycoside prescribing. Population-based genetic screening offers the potential for early carrier identification; however, long-term audiological surveillance, economic viability, and ethical considerations remain important areas for future investigation.

**Conclusion:**

Aminoglycoside-induced hearing loss fulfills the criteria for preventive screening, including a latent preclinical period, available m.1555A>G genotyping tools, and an actionable aminoglycoside avoidance. Although screening appears feasible and early data support the validity of available tools, the clinical utility remains uncertain. Genetic screening may be considered as either a universal component of NBHS programmes or a targeted strategy informed by risk stratification. (family history, NICU admission, aminoglycoside predisposition). Further research on the clinical utility and effectiveness of screening methods is needed to inform neonatal aminoglycoside policy.

## Highlights

- Neonates with mitochondrial mutation m.1555A>G are at risk of aminoglycoside induced sensorineural hearing loss, which is an irreversible, dose-independent injury.
- The median frequency of m.1555A>G is 0.2% (range:0%–0.3%); carriers exposed to aminoglycosides can develop delayed-onset sensorineural hearing loss which may be missed by newborn hearing screen (NBHS).
- Both universal and targeted, risk-stratified genetic screening strategies are viable implementation models; prospective validation studies are needed to establish their operational feasibility, clinical utility and cost-effectiveness.

## 1 Introduction

Aminoglycosides are the most prescribed antibiotics in neonatal intensive care units (NICUs) for treating presumed or culture-proven infections (1, 2). They are potent bactericidal agents that work by inducing ribosomal mistranslation and abnormal protein synthesis. Their efficacy is concentration-dependent, and therapeutic drug levels are monitored to reduce the risks of nephrotoxicity and ototoxicity (3).

While nephrotoxicity is often reversible, aminoglycoside-induced ototoxicity (AIO) is an irreversible injury, resulting in sensorineural hearing loss (SNHL). AIO can manifest as an immediate-onset idiosyncratic reaction or as delayed-onset SNHL, usually bilateral, symmetric high-frequency impairment, and sometimes progressive (3–5). Notably, in individuals with certain mitochondrial DNA mutations, a single therapeutic dose of aminoglycoside (AG) is sufficient to cause harm (6).

The recognition of AIO is profoundly complex in neonates due to their distinct physiology. Preterm infants are particularly vulnerable; immature cochlea, disrupted in-utero maturation of auditory pathways, frequent exposure to noxious stimuli such as noise & ototoxic medications intricately interplay (7). An underlying genetic predisposition further compounds this risk.

Mutations in the mitochondrial 12S rRNA gene (MT-RNR 1) encode mutant 12S rRNA that structurally resembles the bacterial 16S ribosomal subunit, the binding site of aminoglycosides. Thus, aminoglycosides accumulate in tissues rich in mitochondria, disrupting protein synthesis and causing cytotoxic injury. Cochlear hair cells, with a high mitochondrial density, are susceptible to AG-induced cochlear dysfunction i.e., AG-induced hearing loss (AIHL). Three single-nucleotide variants (SNVs) or point mutations of the MT-RNR1 gene, namely m.1555A>G, m.1095T>C, and m.1494C>T, are well-studied to be associated with AIHL (5, 6, 8).

The mechanism of AIHL in preterm infants with MT-RNR1 mutations is postulated by a double-hit model, in which loud NICU noise causes excessive stimulation of basal cochlear hair cells, opening mechanoelectrical transduction (MET) channels—one-way channels that result in increased intracellular accumulation of AG in hair cells. Subsequently, mutant 12S ribosomal subunits (which mirror bacterial 16S rRNA) misdirect AG to bind to human cochlear mitochondria resulting in cytotoxic injury & apoptosis of hair cells leading to irreversible cochlear dysfunction (3, 7).

Mitochondrial DNA is inherited exclusively through the maternal lineage, and pathogenic variants may occur as homoplasmy (all copies mutated) or heteroplasmy (coexistence of mutant and wild-type copies), with heteroplasmy levels varying across different cells, tissues and organs. Homoplasmic m.1555A>G was the earliest pathogenic MT-RNR 1 mutation described in families with maternally inherited AIHL and in a large Arab Israeli pedigree with non-syndromic hearing loss (NSHL) (9). Subsequent literature reported its incidence between 2%–20% in familial NSHL and 10%–30% in AIHL, more frequent among Chinese, Japanese, and Spanish ethnicities. Considerable heterogeneity in the clinical expression of the genotype (e.g., age of onset, severity of HL) was observed across study populations (3, 5, 7, 10). Although m.1555A>G is the primary genetic susceptibility factor, it is not independently causative of hearing loss. The phenotype is modulated by environmental triggers such as aminoglycosides, noise, heteroplasmy levels, mitochondrial haplogroups, and nuclear modifier genes (3, 7, 10).

The Clinical Pharmacogenetics Implementation Consortium (CPIC) states that AG should be avoided in individuals with an MT-RNR1 mutation due to the associated risk of AIO unless the high risk of permanent hearing loss is outweighed by the severity of the infection and safe or effective alternative therapies are not available (11). However, aminoglycosides remain the recommended first-line antibiotics in NICUs (12, 13).

Detection of m.1555A>G carrier status prior to AG treatment can potentially avert irreversible HL. Historically, allele-specific amplification by polymerase chain reaction (PCR) and Sanger sequencing were standard methods for genetic testing and their widespread use was limited by centralised expertise, time and cost. However, molecular genetics has advanced exponentially in the last three decades, expanding the realm of genetic testing to impact clinical care (14, 15). Recently, the Pharmacogenetics to Avoid the Loss of Hearing (PALOH) trial demonstrated the feasibility of using the Genedrive® MT-RNR1 assay as a rapid point-of-care test (POCT) for neonatal screening of the m.1555A>G variant prior to aminoglycoside administration (16).

There is an urgent need to synthesize the existing evidence on m.1555A>G AIHL and to evaluate current diagnostic strategies to inform effective approaches for safeguarding auditory health in newborns. For practicing neonatologists, key questions include: How prevalent is m.1555A>G? What is the strength of its association with AIHL? Is it feasible to screen neonates before prescribing aminoglycosides? Should screening be universal or limited to high-risk groups? Is the continued use of aminoglycosides without genotyping justified?

This empirical, integrative narrative review focused on mapping the scholarly landscape of m.1555A>G and AIHL in neonatal clinical practice through a conceptual framework based on the Wilson and Jungner screening principles, structured around three core domains of public health screening (17, 18): Disease/condition—the frequency of the m.1555A>G mutation and AIHL in neonates; Test—the analytical validity and clinical utility of genetic tests for m.1555A>G; and System—the operational framework, economic evaluation, clinical benefits, and ethical complexities of integrating genetic screening into routine neonatal care.

## 2 Methods

To ensure methodological rigor of this integrative review, Whittemore and Knafl's framework (19) was used, comprising five stages—problem identification, literature search, data evaluation, data analysis, and presentation of results.

### 2.1 Literature search

A comprehensive literature search was conducted in Cochrane Library, PubMed, MEDLINE, Embase, and ClinicalKey from 2000 to August 8, 2025. The search strategy was ((((neonates) OR (newborn)) AND (((((MT-RNR1) OR (12s rRNA gene)) OR (M1555A>G)) OR (m.1555A>G)) OR (A1555G))) AND ((aminoglycoside) OR (aminoglycosid*))) AND ((((((hearing loss) OR (hearing impairment)) OR (sensorineural hearing loss)) OR (sensorineural hearing*)) OR (deafness)) OR (ototoxicity)). Figure 1 shows the PRISMA flow chart with the search trail details.

**Figure 1:**
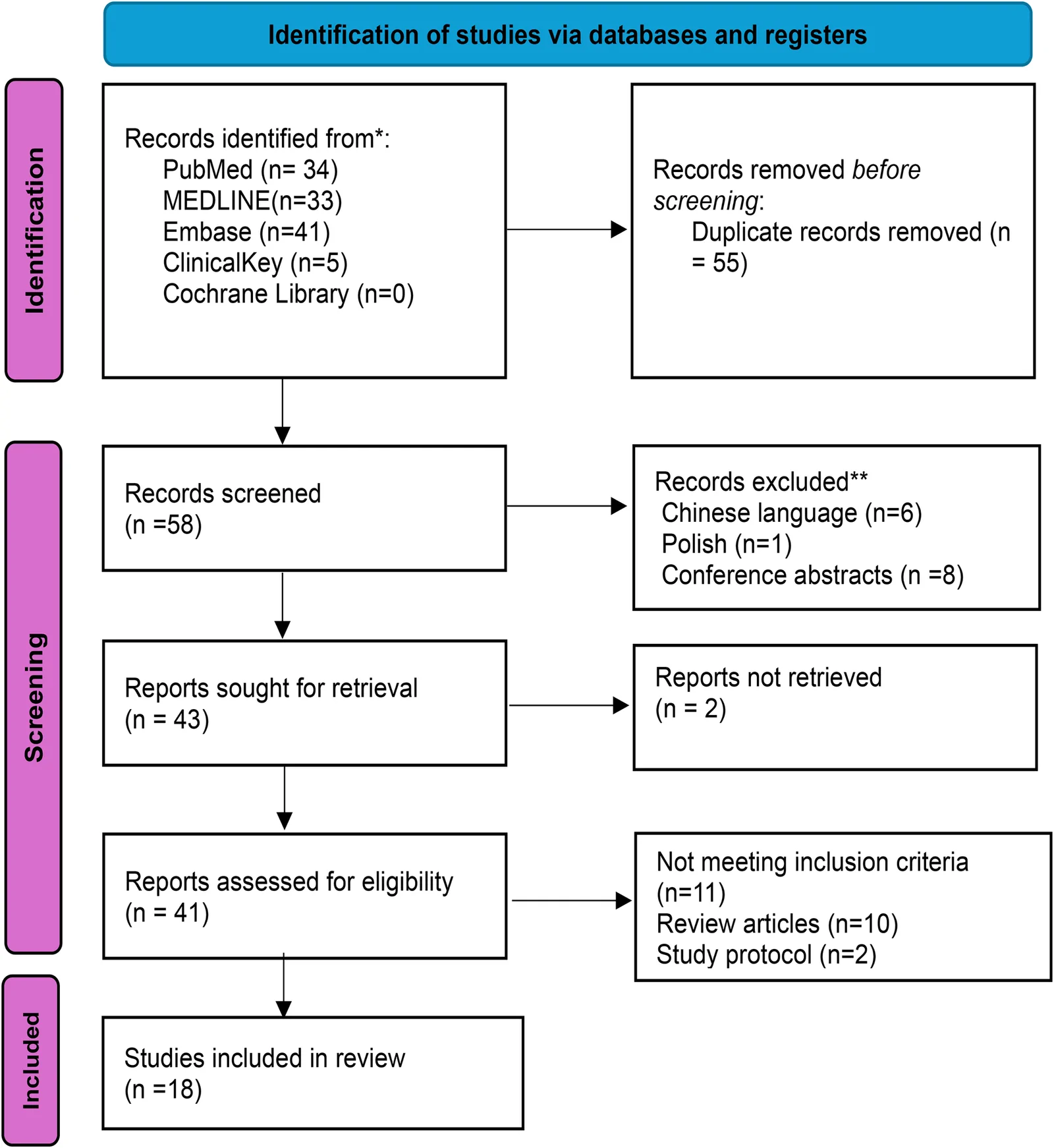
PRISMA flowchart of literature search showing the systematic literature selection process. Brackets denote the number of records (*n*) analysed at each discrete assessment step. Specific criteria and quantifiable counts for the exclusion of full-text articles during the final eligibility phase are explicitly detailed in the right-hand panel boxes PRISMA, Preferred Reporting Items for Systematic Reviews and Meta-Analyses.

Studies were included if they investigated the MT-RNR1m.1555A>G variant, reported primary data from neonatal populations, were published in peer-reviewed English-language journals, and were available in full text. Studies that did not include m.1555A>G or the neonatal population, conference abstracts, grey literature, opinion papers, and case series were excluded.

### 2.2 Data evaluation and analysis

Eighteen primary studies satisfied the eligibility criteria and were included in the review (16, 20–36). Full-text articles were reviewed, and key data were systematically extracted using a standard abstraction form. The constant comparison method was applied throughout data reduction and analysis using tables of discrete variables, coded data visualization boards, and concept matrices to identify common themes and unique features. Study-specific prevalence estimates were calculated as n/N (frequency of m.1555A>G-positive individuals divided by the total number screened) with corresponding 95% CIs derived using the Wilson score method. Owing to heterogeneity among studies, prevalence estimates were summarized using the median prevalence across studies. Risk of bias was evaluated using the Joanna Briggs Institute (JBI) Critical Appraisal Tools, with study-specific checklists applied for cohort, cross-sectional, and case-control studies (Supplementary material).

## 3 Results

A total of 18 studies were included and are presented in chronological order, with their key characteristics summarized in Table 1. These studies employed diverse methodologies and were published across a range of academic journals, reflecting the heterogeneity noted above.

**Table 1:** Key characteristics of included studies.

Study (Author, year)	Purpose of the study	Methodology/Study period	Subjects/Setting	Key results
Gravina et al., 2007 (20)	Evaluate prevalence of m.1555A>G & 35delG mutation, most associated with NSHL in the Argentinian population	Single-centre cross-sectional genetic prevalence study.^a^	Random anonymous blood samples of 712 healthy blood donors & 330 DBS of neonates tested using PCR- RFLP.	Nil cases of m.1555A>G. Frequency of 35delG- 1/65, 1.54% (95% CI: 0.64%–2.45%)
Maniglia et al., 2008 (21)	Investigate prevalence of m.1555A>G in patients with NSHL and its association with AG exposure	Single centre cross-sectional case study, Sept–Oct 2006	Blood samples of 27 cases of HL & umbilical cord blood samples of 100 newborn infants were tested by PCR-RFLP.	Nil cases of m.1555A>G.
Johnson et al., 2010 (22)	Investigate association between MT-RNR1 gene mutations, AG exposure, and HL in low-birth-weight NICU infants	Multi-centre prospective case cohort study, 2005–2007	DNA Sequencing/ PCR of DBS from cord blood or serum of infants (*n* = 436) collected within 48 h of life. Staged NBHS (OAE & ABR) to assess hearing outcomes	378/436 (87%) exposed to Gentamicin during NICU stay. 256 (60%) had hearing assessment. 3 carriers of m.1555A>G & 1m.1494T>C detected. All had AG exposure; One infant with m.1555A>G failed NBHS.
Nivoloni et al., 2010 (23)	Introduce genetic screening to NBHS program to widen diagnostic potential & reduce time to etiologic diagnosis	Prospective cohort study,2003–2006	8974 random newborn infants underwent NBHS & screening for 35delG & 2 MT-RNR1 mutations using DBS & PCR. Genetic screen extended to those who failed NBHS	Genetic screen identified 91 cases; 4 homozygous 35delG (failed NBHS), 3m.827A>G (passed NBHS) & 84 GJB2 35delG carriers (passed NBHS). None had m.1555A>G.
Ealy et al., 2011 (24)	Determine the relative prevalence of 5 variants of MT-RNR1 [A1555G, C1494T, T1095C, 961delTþC(n), A827G] in NICU & general Iowan population	Single centre-cross-sectional prevalence study.^a^	DNA samples from 703 NICU infants & 1473 blood bank samples of de-identified Iowans were screened by Multiplex genotyping assay	No difference in frequency between neonates & general population (1.85% vs. 1.83%). Commonest variants—961delTþC(n) & A827G (0.78% each). Frequency of m.1555A>G—0.23%, 2m.1555A>G carriers with >1 week of gentamicin exposure passed NBHS.
Wang et al., 2011 (25)	Evaluate efficacy of combined NBHS & genetic screening program across 11 provinces of China by Newborn Gene Concurrent Hearing Screening (NGCHS)Study group	Multi-centre large- scale epidemiological study, 2007–2009	DBS from umbilical cord blood samples of newborns (*n* = 14,913) were screened for GJB2, SLC26A4 & MT-RNR1 variants by PCR & direct sequencing along with 2-staged NBHS	Genetic screen positive rate—2.05% (306/14,913). 85% (261/306) passed NBHS. 25 had causative mutations (1.68 per 1,000) & 281 non-causative heterozygous carriers. 18/25 were m.1555A>G cases (passed NBHS). Extended pedigree evaluation of 16 cases of m.1555A>G identified 11 (7 females) deaf subjects & 123 carriers (77 females)
Göpel et al., 2014 (26)	Evaluate if AG exposure in preterm VLBW infants with m.1555A>G mutation resulted in failed NBHS. To determine feasibility of maternal genetic screen to prevent AG use in carrier neonates.	Multi-centre prospective cohort study, 2003–2012	Paired mother-infant buccal samples or umbilical cord tissue (*n* = 7,056) of VLBW infants from 46 NICUs in the German Neonatal Network were tested by a custom genotyping assay	AG treatment in m.1555A>G-carriers was significantly associated with failed NBHS. (OR: 1.26, 95% CI: 1.07–1.49, *p* = 0.0058). Low birth weight was the most important predictor of failed NBHS (OR: 0.91, 95%CI: 0.88–0.94 per 100 g additional birth weight). Genotyping of maternal DNA was successful in 3,002 of 3,070 samples (97.7%) and 100% corroborative with neonatal results.
Li et al., 2015 (27)	Feasibility of genetic screening for deafness using cord blood samples & assess if it complements NBHS.	Single centre prospective cohort study, 2010–2011	646 newborns underwent combined NBHS and genetic screening for 20 hotspots in 4 common HL genes^b^ using Mass Array technology- multiplex PCR & Sequencing.	NBHS fail rate- 1.7% (11/646) & Genetic screening detection rate—4.5% (29/646) 2 cases of m.1555A>G (0.3%).337/635 (53%) -suspected pathogenic mutations. Provided epidemiology of mutations prevalent in Jinan, China to tailor the local genetic screening panel.
Soini et al., 2017 (28)	Estimate the population prevalence of 3 MT-RNR1 variants in Finland.	Single centre epidemiological study,1973–2012	Buccal, blood or umbilical cord samples from newborn infants (*n* = 813, 624 preterm,189 term) were genotyped for m.1555A>G, m.1494C>T and m.1095T>C by PCR-RFLP with quantification of heteroplasmy levels.	1 case of m.1555A>G—term infant with 81% heteroplasmy level, passed NBHS and audiometry tests at 5 & 7 yr. of age. Mother had m.1555A>G at 68% heteroplasmy, normal hearing. Nil cases of m.1494C>T and m.1095T>C.
Dai et al., 2019 (29)	To demonstrate feasibility of combined hearing & genetic screening to improve detection of HL in newborns and evaluate the implemented program in a megacity.	Population-based cohort study, 2013–2014	180,469 newborn infants across 132 maternity centres in Beijing received concurrent NBHS and genetic screening using DBS & Array Kit for 9 variants in 4 genes^b^ at designated hospitals, free of cost. Follow-up on hearing and habilitation of cases until 5 years by telephone survey.	NBHS fail, rate- 1% (*n* = 1,915). Genetic screen detection rate -4.5% (*n* = 8,136). PPV for NBHS 18%.PPV for genetic screen >90%. 405/409 with MT-RNR1 (m.1555A>G or m.1494C>T) passed NBHS. Range of heteroplasmy -1.85–89.14%. GJB2 & SLC26A4 account for about 66% of genetic HL. Follow-up rate for MT-RNR 1 carriers was 35%.@2–3yrs.
Liu et al., 2019 (30)	Investigate the genetic aetiology of congenital HL, frequency of genetic mutations in newborn infants and its correlation with NBHS results.	Single centre-cross-sectional study, 2014 & 2016	1,315 neonates who failed NBHS & 1,000 random neonates were screened for 101 mutations in 18 HL-related genes using DBS by Multiplex PCR and Next generation sequencing (NGS).	Frequency of mutations higher in NBHS-fail group vs. controls (37.0% vs. 19.4%, *p* < 0.0001). GJB2 c.109G>A, the most frequent, significantly higher in NBHS-fail group (16.8% vs. 7.6%, *p* < 0.0001). MT-RNR 1 mutation frequency comparable between groups (0.84% vs. 0.60%; *p* = 0.68).
Zhu et al., 2021 (31)	Assess feasibility & utility of a modified genetic & hearing screening program for newborn infants.	Multi-centre population-based cohort study, (2016–2020)	32,512 neonates underwent a 4-staged NBHS and genetic screen for 15 variants in 4 common genes using DBS samples by Microarray Kit (*n* = 32,512) with 5-year audiology follow-up.	Prevalence of congenital HL 4.4 per 1,000 (95% CI, 3.7–5.1per1000). Prevalence of genetic variations—4.0% (95% CI, 3.8%–4.2%). Limited genetic screen detected 31 HL cases missed by NHS (21.8%). Expanded genetic screen for 228 genes identified 17 additional HL cases (18.5% detection rate).
Chant, Bitner-Glindzicz and Marlow, 2022 (32)	Study individual & cumulative risk factors for HL during neonatal period.	Case-control study, (2009–2013)	57 ex-preterm infants (<32 wks. gestation) with HL compared with 180 controls. Genotyping m.1555A>G done using buccal swabs of subjects & confirmed by DNA Sequencing.	Physiological lability [OR: 1.15 per day (95% CI: 1.05–1.27)] and ototoxic medications [OR: 1.23 per day of exposure (95% CI: 1.1–1.37)] in NICU were strongly associated with HL. One case of m.1555A>G in control group with AG exposure in NICU developed pure tone HL at 3-yr. follow up.
Huang et al., 2022 (33)	To evaluate the performance of non-invasive prenatal testing (NIPT) for detecting m.1555A>G and m.1494C>T mutations and to analyse the correlation of haplogroups with m.1555A>G.	Single centre cross-sectional diagnostic accuracy study	Fetal cell-free DNA from 76,842 maternal NIPT samples including 327 paired with DBS of neonates genotyped using PCR & Sequencing.	PPV of NIPT sample- 90.8% for m.1555A>G. Prevalence of m.1555A>G (0.22%) & m.1494C>T (0.02%) was comparable to contemporaneous data (*p* > 0.05). In 327 paired mother-infant samples, NIPT corroborated with DBS results. m.1555A>G was enriched in sub- haplotype D5 [*p* = 0, OR = 4.6706 (2.81–7.78)]
McDermott et al., 2022 (11)	Develop a rapid point-of-care test (POCT) to genotype m.15555A>G & evaluate feasibility of its use in acute neonatal care to avoid AIHL. Pharmacogenetics to Avoid the Loss of Hearing (PALOH)trial.	Multi-centre prospective implementation trial,	Buccal swabs of 751 NICU infants were subject to POCT for m.1555A>G prior to receiving gentamicin using Genedrive- MT-RNR1 assay platform & melt peak analysis.	Preclinical validation of Genedrive- MT-RNR1 assay (n-159) demonstrated a 100% sensitivity (95% CI, 93.9%–100.0%) & specificity (95% CI, 98.5%–100.0%).80% (424/526) of infants who received antibiotics were genotyped. No difference in mean (SD) time to antibiotic therapy [55.87 (22.56) vs. 55.18 (23.82)] before and after introduction of POCT.
Kun et al., 2023 (34)	Explore the distribution & prevalence of 15 known HL mutations, the genotype-phenotype correlation &value of 1-year clinical follow-up.	Multicentre prospective cohort study, (2019–2021)	DBS from 77,647 neonates recruited from 38 community centres were analysed for 15 mutations using Microarray platform.	(i) 2,369/77,647 (3.05%) carried at least one HL variant. GJB2 (1.48%) and SLC26A4 (1.07%) were the most common. (iii) 234 neonates (0.30%) carried MT-RNR1 mutations; all passed NBHS. Only 49.18% (1,165/2,369) of positive cases attended 1-year follow-up.
Duan et al., 2025 (35)	To elucidate the frequency of MT-RNR1 mutations from the integrated universal screening program and describe its phenotypes, heteroplasmy, correlation with haplogroups and medication history.	Population-based cohort study, (2013–2014)	180,458 neonates underwent combined hearing and genetic screening by NGS. Matrilineal family members of probands were tested and follow-up of hearing outcomes described.	409 carriers of MT-RNR1 (382m.1555A>G & 27–m.1494C>T) detected. 76% of m.1555A>G were homoplasmic. Follow-up rate until 2 years -35% (142/409). 95% with positive family history carried homoplasmic m.1555A>G. 26% mothers of probands had HL. No statistical difference in hearing between vaccinated and unvaccinated mothers of probands vs. controls. Haplotype D associated in 50.8% carriers.
Shi et al., 2025 (36)	Investigate risk factors for HL in high-risk neonates admitted to NICU and analyse the genetic/audiological profiles of prevalent mutations.	Observational study of risk stratified cohorts within the universal NBS program, (2022–2023)	High-risk cohort: 443 NICU infants who failed NBHS underwent deafness sequencing panel for 20 mutations across 4 common HL genes^b^. NICU risk factors were retrospectively analysed. NBHS included AABR, DPOAE & 1,000 Hz Acoustic Imminence. Larger cohort: 14,863 newborn infants underwent combined NBHS & genetic screen for 20 mutations using DBS samples & NGS.	50% of high-risk group failed diagnostic hearing tests. Preterm birth (*p* < 0.05) by failed AABR, hyperbilirubinemia (*p* = 0.045) & advanced maternal age (*p* = 0.008) by failed AI correlated with HL. Diagnostic yield of genetic screen in high-risk group with HL was 21.2% (7/33) vs. 3.3% in the larger cohort (497/14,863). MT-RNR 1 mutations accounted for 15% (74/497) of positive cases.

a Study period unclear NSHL, Non syndromic hearing loss; NBHS, Newborn hearing screen; DBS, Dried blood spot; VLBW, very low birthweight.

b 4 common HL genes, GJB2, GJB3, SLC26A4, and MT-RNR1; PCR, Polymerase chain reaction; RFLP, Restriction fragment length polymorphism; CI, confidence interval; SD, standard deviation.

The included evidence was entirely observational and markedly heterogeneous in design, consisting of cohort, cross-sectional, case-control, and feasibility studies. Nine studies involved diverse ethnic groups within the Chinese population (25, 27, 29–31, 33–36), five focused on the North & South American population (20–24), and four studied individuals of European descent (16, 26, 28, 32).

### 3.1 Prevalence of m.1555A>G

Among the included studies, five were primarily epidemiological studies (20, 21, 24, 28, 30) and eight were population-based cohort studies that investigated universal genetic screening for hearing loss and reported the frequency of mutations associated with NSHL among other outcomes (23, 25, 27, 29–31, 34–36). All included studies reported the frequency of m.1555A>G either alone or along with other MT-RNR1 gene polymorphisms or nuclear mutations in known deafness-associated genes such as GJB2 (Connexin 26), GJB3 (Connexin 31), and SLC26A4.

To address heterogeneity in study populations, prevalence estimates were summarized separately for population-based or epidemiological newborn cohorts and for selected neonatal cohorts. Across population-based or epidemiological cohorts, the median study-level frequency of m.1555A>G was 0.2% (range 0%–0.3%; Table 2), corresponding to approximately 1 in 500 newborns in the populations studied. Prevalence in selected cohorts, including NICU, LBW/VLBW, aminoglycoside-exposed, and feasibility-study populations, is presented separately in Table 3.

**Table 2:** Prevalence of m.1555A>G among newborn cohorts.

Study (Author, year)	Country/Ethnicity	Study population	n/N	Frequency of m.1555A>G, %, (95% CI)^a^	Reference
Gravina et al., 2007	Argentina	Healthy blood donors (*n* = 712) & neonates (*n* = 330)	0/330	0 (0–1.1)	(20)
Maniglia et al., 2008	Bazil	Control newborns who passed NBHS	0/100	0 (0–3.62)	(21)
Nivoloni et al., 2010	Brazil	Newborns	0/8,974	0 (0–0.04)	(23)
Wang et al., 2011	China	Newborns	18/14,913	0.1 (0.08–0.19)	(25)
Li et al., 2015	China	Newborns	2/646	0.3 (0.08–1.12)	(27)
Soini et al., 2017	Finnish	Newborns	1/813	0.1 (0.02–0.69)	(28)
Dai et al., 2019	China/Han	Newborns	382/1,80,469	0.2 (0.19–0.23)	(29)
Liu et al., 2019	China	Newborns	3/1,000	0.3 (0.10–0.87)	(30)
Zhu et al., 2021	China/Han	Newborns	2/32,512	0.01 (0.0–0.02)	(31)
Kun et al., 2023	China	Newborns	221/77,647	0.3 (0.25–0.32)	(34)
Duan et al., 2025	China/Han	Newborns	382/1,80,458	0.2 (0.19–0.23)	(35)
Shi et al., 2025	China	Newborns	31/14,863	0.2 (0.15–0.30)	(36)

a Wilson's 95% confidence intervals.

**Table 3:** Frequency of m.1555A>G in selected neonatal populations.

Study (Author, year)	Study population	Purpose of study	n/N	Frequency of m.1555A>G, %, (95% CI)^a^	Reference
Johnson et al., 2010	LBW NICU infants, USA	Frequency & relationship of MT RNR 1 gene mutations & AG exposure or other risk factors for HL	3/436	0.7 (0.23–2.02)	(22)
Ealy et al., 2011	USA	NICU infants	2/703	0.3 (0.03–1.0)	(24)
Göpel et al., 2014	VLBW NICU infants, German Neonatal Network	Association between AG exposure in m.1555A>G carriers and failed NBHS	12/7,056	0.2 (0.10–0.30)	(26)
Chant, Bitner-Glindzicz and Marlow, 2022	Control group- Ex-<32 weeks NICU infants, UK	Describe cumulative risk factors for HL in neonatal period	1/180	0.6 (0.01–3.06)	(32)
Huang et al., 2022	Paired NIPT^b^—neonatal genetic data, China	Evaluate the performance of NIPT for detecting m.1555A>G	2/327	0.6 (0.17–2.19)	(33)
McDermott et al., 2022	NICU infants who required AG, UK	Feasibility of POC^c^ genotyping in the acute neonatal setting	3/424	0.7 (0.24–2.07)	(16)

a Wilson's 95% Confidence intervals LBW, Low birth weight; VLBW, Very low birth weight; AG, aminoglycoside.

b NIPT, Non-invasive prenatal testing.

c POC, point-of-care.

Of note, Maniglia et al. investigated the prevalence of the A1555G mutation in 27 patients with SNHL with and without aminoglycoside use and 100 control newborn infants who passed the newborn hearing screen (NBHS) and did not detect any carriers (21). In the cross-sectional epidemiological study by Liu et al., the frequency of mitochondrial mutations did not differ significantly between neonates in the NBHS pass and fail groups (0.837% vs. 0.600%; *p* = 0.679) (30). Apart from Huang et al., the studies presented in Table 3 were conducted predominantly in high-risk NICU populations and were constrained by small sample sizes and the observed median frequency was 0.6% (range 0.2%–0.7%). Huang et al. demonstrated the diagnostic accuracy of maternal non-invasive prenatal testing (NIPT)-based screening, reporting prevalence estimates consistent with those of contemporaneous studies (25, 29).

### 3.2 Association of m.1555A>G and failed NBHS

Five studies examined the association between m.1555A>G, AG exposure, duration of therapy, gestational immaturity and NBHS outcome (22, 24, 26, 32, 35). Three studies found no association between AG exposure and NBHS outcome (22, 24, 35). A study from the German Neonatal Network noted a significant but weak association between AG exposure and failed NBHS in VLBW infants with m.1555A>G (OR: 1.26, 95% CI: 1.07 to 1.49, *p* = 0.0058) (26). In a retrospective case-control study of 57 ex-very preterm infants (<32 weeks' gestation) with hearing loss and 180 matched controls, hearing loss was independently associated with physiological instability during the neonatal period (OR: 1.15, 95% CI: 1.05–1.27 per day) and cumulative exposure to ototoxic medications, including gentamicin, vancomycin, and furosemide (OR: 1.23, 95% CI: 1.10–1.37 per day). The m.1555A>G variant was identified in one control infant who had been exposed to gentamicin during the neonatal period and passed NBHS. Although hearing screening at 2 years of age was normal, pure-tone hearing impairment was detected at the 3-year follow-up (32).

### 3.3 Genotyping methods

Rapid advances in molecular diagnostics have led to the development of increasingly sophisticated tools for the detection of MT-RNR1 gene mutations. While earlier studies relied on low-throughput methods such as polymerase chain reaction (PCR) and Sanger sequencing (22–25), subsequent research employed advanced multiplex assay/microarray platforms (20, 21, 32) and, more recently, high-throughput Next-generation sequencing (NGS) (31, 35, 36).

The PALOH trial demonstrated the analytical validity of a POCT based on rapid thermocycling and melt peak analysis for genotyping. Using buccal samples containing a minimum of 16 cells, the assay achieved 100% sensitivity (95% CI: 93.9%–100%) and 100% specificity (95% CI: 98.5%–100%), with results available within 26 min (16). However, recruitment challenges limited the study largely to a single-centre experience, potentially restricting the generalizability of its findings.

### 3.4 Genetic screening strategies

Several screening strategies for the m.1555A>G variant have been explored, including both targeted and population-based approaches. The feasibility of integrating universal genetic screening with NBHS program was first evaluated by the Newborn Gene Concurrent Hearing Screening (NGCHS) Study group (25), and its performance and benefits were later reported from various regions of China (27, 29–31, 34–36). Table 1 highlights key features of these studies. Universal genetic screening employed assay panels with different combinations of mutations in the core NSHL genes—*GJB*2, *GJB*3, *SLC*26*A*4, and *MT-RNR*1. Integrated NBHS programs with concurrent universal genetic screening led to early detection and intervention for individuals with pathogenic mutations and offered genetic counselling, pedigree analyses, audiological surveillance and health alert cards to avoid aminoglycosides for MT-RNR1 mutation carriers. Long-term follow-up through 2 years of age was achieved in fewer than 50% of identified carriers (29, 34, 35).

Duan et al. described the phenotypes of MT-RNR1 mutations, heteroplasmy levels, and their correlation with haplogroups in a large population-based study (*n* = 180,458) of the integrated NBHS program (35). Among 382 m.1555A>G carriers, 76% were homoplasmic, and 96% of those with a positive family history carried the homoplasmic variant.

Novel antenatal screening strategies for the m.1555A>G variant have been explored in selected populations, with studies demonstrating the feasibility of both maternal buccal sampling and NIPT for early identification of at-risk neonates. Göpel et al. demonstrated the operational feasibility and diagnostic accuracy of antenatal non-invasive screening for the m.1555A>G variant using maternal buccal samples (26). The study by Huang et al. demonstrated the feasibility of detecting the m.1555A>G variant in fetal cell-free DNA (cfDNA) obtained from maternal blood samples collected for non-invasive prenatal testing (NIPT) (33). Both methods showed 100% agreement between maternal and neonatal genotypes.

## 4 Discussion

Overall, the m.1555A>G variant is rare, although its prevalence varies considerably across regions, likely reflecting differences in ethnic composition and population genetic structure. Preterm infants are at increased risk of hearing loss due to multiple perinatal factors, and m.1555A>G carrier status may confer additional susceptibility to auditory impairment. Because AIHL can present after the neonatal period, it may not be detected through routine NBHS, emphasizing the importance of early carrier detection and longitudinal audiological monitoring. Advances in molecular diagnostics have driven parallel developments in epidemiological research and screening strategies.

Evidence from small cohort studies suggests that targeted screening for the m.1555A>G variant is both feasible and accurate in selected populations, including VLBW infants and pregnant women. Evidence from large population-based studies, predominantly conducted in China, suggests that universal genetic screening for congenital hearing loss, including the m.1555A>G variant, can be successfully implemented alongside NBHS and may enhance early detection of at-risk infants.

The findings of this review align with several key principles of preventive health screening: an identifiable population at increased risk of AIHL can be detected before disease onset through m.1555A>G genotyping, and a clear preventive intervention, avoidance of aminoglycoside exposure, is available for carriers. Accordingly, the evidence warrants evaluation within the Wilson-Jungner framework to determine the suitability of systematic m.1555A>G screening programmes.

### 4.1 Prevalence of m.1555A>G

Overall, m.1555A>G is a rare mutation, with prevalence varying by region according to ethnic composition, as shown in Table 2. Notably, small epidemiological studies among South American populations reported no cases (20, 21, 23). Several large population-based studies from China consistently reported a carrier frequency of approximately 0.2%, with narrow confidence intervals indicating high precision and reliability of the prevalence estimates (29, 34–36). Higher carrier frequencies, of up to 0.7%, were reported among high-risk infants. Nevertheless, the corresponding prevalence estimates were less precise, as indicated by wider confidence intervals, likely attributable to limited sample sizes (Table 3). Despite increasing evidence from several regions, there remains a notable lack of epidemiological data from Africa, Australia, Southeast Asia, and the Indian subcontinent, limiting the generalisability of current prevalence estimates.

Nevertheless, the median study-level frequency of 0.2% (0%–0.3%) aligns with the findings of a recent systematic review (31 studies) by Gaffar et al. on the pharmacogenetic determinants of AIO. The A1555G variant was the most frequently reported, with a prevalence ranging from 0% to 17% in cohorts with reported AIO and 0% to 0.2% in healthy subjects. Aminoglycoside exposure was associated with a lower age of disease onset and a high penetrance of the phenotype (up to 100%, level III–IV evidence) (37).

An earlier systematic review and meta-analysis of 45 studies estimated a global pooled prevalence of 2% (95% CI: 1%–4%; *I*^2^ = 99%) for three variants of MT-RNR1 mutations, including m.1555A>G. More than half of the included studies were conducted among hearing-impaired cohorts or family clusters. Prevalence estimates across different populations varied widely: Africa 0.9%–2%, Asia 0.09%–17%, Poland 0.4%, America 1.85%, China and Spain 17%. The penetrance of the mutation following AG exposure was approximately 60% in Korean, Spanish, and Arab-Israeli populations, and was significantly lower (4%–18%) in Chinese cohorts (38).

MT-RNR1 mutations typically present in one of three ways: sporadic NSHL, familial clusters of NSHL with matrilineal inheritance, or as reported AIHL. Epidemiological studies have consistently observed a tenfold or higher prevalence of m.1555A>G in NSHL cohorts compared to 0.2% in the general population (8, 10, 37). And the mutation frequency was further enriched in NSHL cohorts reporting aminoglycoside exposure (8, 10, 37, 38).

These observations are well-consolidated in a recent systematic review and meta-analysis (97 studies, *n* = 31,013) on the global prevalence of m.1555A>G in NSHL (39). The variant was detected at a median frequency of 1.69% (range: 0–27.14%; mean: 3.37%) with a statistically significant association with NSHL (OR=14.26, 95% CI: 9.13–22.26, *P* < 0.01). It was more prevalent among post-lingual vs. pre-lingual HL (4.35% vs. 1.47%), familial vs. sporadic cases (9.19% vs. 2.69%), positive history of AG exposure vs. without (13.11% vs. 2.59%) and in subjects with severe-to-profound HL compared to milder forms.

Based on current evidence, the carrier frequency of the m.1555A>G variant is approximately 1 in 500 neonates. However, large population-based studies across diverse populations are required to improve estimates of regional prevalence. Such data are critical for informing context-specific screening programmes, antibiotic prescribing policies, and other measures to prevent AIHL.

### 4.2 Association of m.1555A>G and AIHL

Interpretation of the association between m.1555A>G, aminoglycoside exposure, and hearing loss is constrained by the available evidence, which is largely based on NBHS outcomes and lacks the longitudinal follow-up required to ascertain delayed-onset AIHL.

The auditory trajectory of m.1555A>G carriers is heterogeneous, varying in both the timing and severity of hearing loss, and exposure to ototoxic agents (AG) may further adversely affect outcomes (10, 37–39). A few population-based studies reported longitudinal audiological surveillance, with substantial attrition during follow-up underscoring the challenges of long-term follow-up in neonatal cohorts (29, 31, 34, 35). Further longitudinal studies are therefore needed to clarify the progression of auditory function and the relationship between *m.*1555*A>G*, aminoglycoside exposure, and hearing loss.

Moreover, AIHL primarily affects the high frequencies and can be missed by NBHS protocols based on otoacoustic emissions (OAE) and automated auditory brainstem response (AABR). These conventional screening tools are designed for a frequency range of 1–4 kHz, and early stages of injury are likely under-estimated (40). Diagnostic audiological evaluation can prove beneficial for the timely recognition of AIHL and should be considered for high-risk neonates.

Another notable limitation is the paucity of data on preterm infants, a population at increased risk of hearing loss. Only four of eighteen studies were conducted exclusively among this high-risk cohort (22, 24, 26, 32). Evidence from these studies suggests that low birthweight, NICU stay, physiological instability, and concurrent ototoxic medication use may compound the risk of hearing loss in neonates. However, larger longitudinal studies are needed to confirm these findings and assess their long-term clinical implications.

The role of other phenotype-modulating factors (such as potential threshold effects, nuclear modifier genes, and environmental triggers) remains under investigation. Although pathogenic MT-RNR1 variants are more commonly homoplasmic, there is currently no established heteroplasmy threshold that reliably predicts clinical expression of the m.1555A>G genotype (29, 35). Robust longitudinal studies are therefore needed to evaluate the contribution of heteroplasmy and other modifying factors, and to better characterise the association between m.1555A>G carrier status, aminoglycoside exposure, and hearing outcomes.

Nevertheless, important evidence gaps remain. Until these are addressed, a cautious clinical approach is justified, and proactive m.1555A>G genotyping may be considered, particularly in neonates at high risk of aminoglycoside exposure.

The primary aim of preventive screening is to identify conditions or susceptibility states before disease onset and to enable the implementation of risk mitigation measures to minimise adverse outcomes. In relation to the disease characteristics domain of the Wilson-Jungner framework, screening for the m.1555A>G variant may be warranted because it detects a genetic susceptibility to AIHL, which can range in severity from mild to profound. Moreover, the estimated carrier frequency of approximately 1 in 500 neonates is comparable to, or higher than, that of several inherited conditions currently included in expanded newborn screening programmes (41, 42).

### 4.3 Genetic tests for m.1555A>G

Population-level genetic screening necessitates a balanced assessment of operational feasibility, analytical validity and ethical considerations. While a detailed discussion of molecular diagnostic technologies is beyond the scope of this review, their current capabilities and rapid advancement merit consideration.

#### 4.3.1 Sampling methods & operational feasibility

Several sampling methods—such as buccal swabs, dried blood spots, cord blood, and fetal cell free-DNA from maternal blood—have demonstrated high concordance with conventional whole-blood DNA analysis (Table 1). Buccal swabs are non-invasive, easy to collect, and provide DNA of comparable or superior quality that remains stable for extended periods at room temperature (43). It is well-suited for large-scale screening as it greatly reduces logistical and economic barriers compared to venous samples and improves participant compliance. Utilizing modern molecular techniques, including droplet digital PCR, NGS, and high-resolution melt profiling, buccal swabs are validated for accuracy and can detect SNVs with thresholds as low as 1% heteroplasmy (43, 44).

Dried blood spot (DBS) sampling was established as a versatile method by the expanded NBS programs, with the introduction of tandem mass spectrometry in the early 2000s. The use of archived DBS for population-based genomic studies using whole-genome sequencing (WGS) is gaining momentum, highlighting their potential to enhance and broaden the scope of preventive screening programmes (41, 44).

#### 4.3.2 Assay tools & analytical validity

As evident in Table 1 and illustrated in Figure 2, rapid advances in molecular diagnostics have improved analytical precision, enabled high-throughput automation, reduced testing costs, and facilitated a shift from complex laboratory-based assays to rapid and accessible point-of-care technologies (44–46).

**Figure 2:**
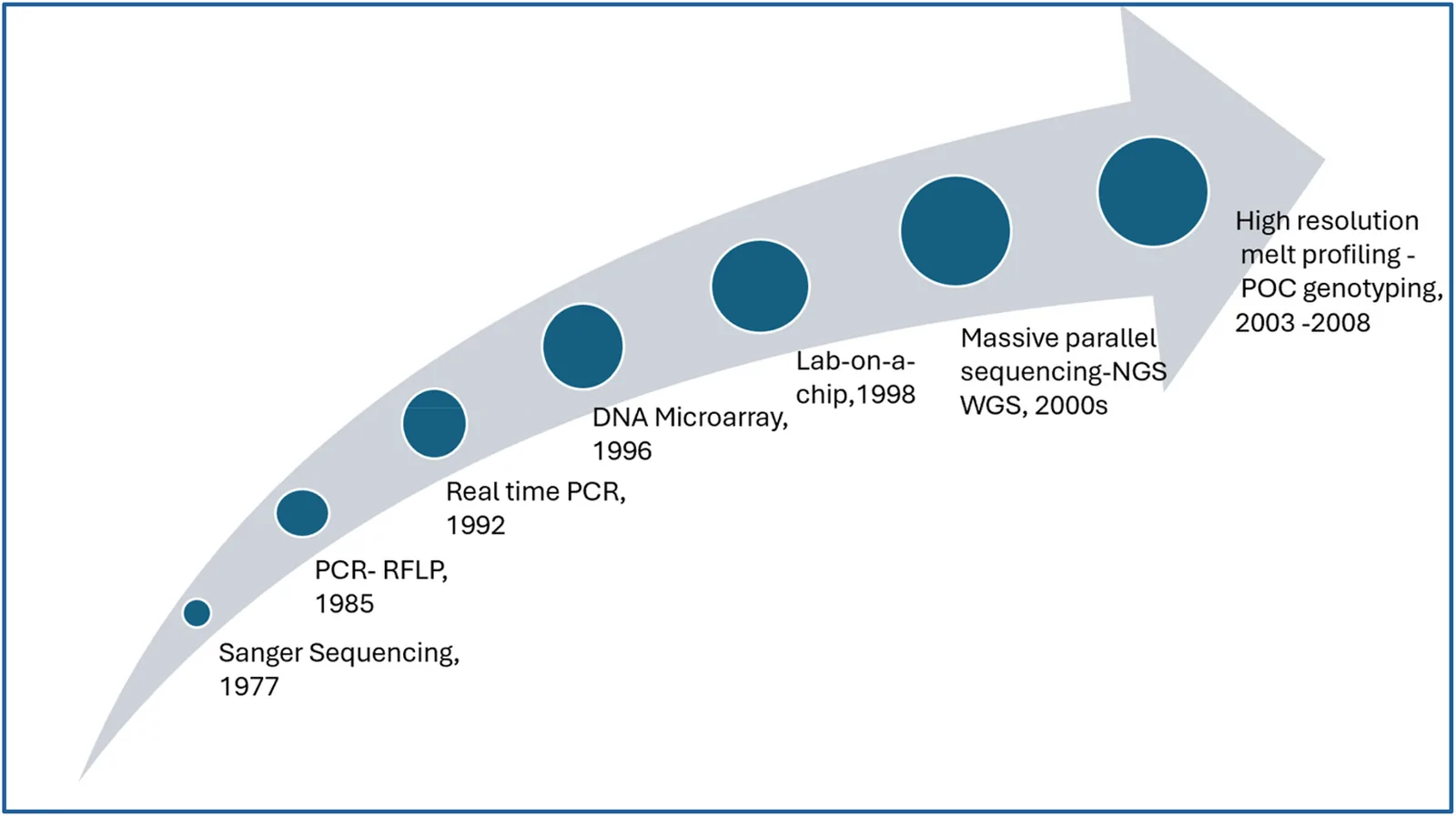
Timeline of technical advances in molecular diagnostic tools showing the rapid evolution of genetic tools between 1977 and 2010. PCR, Polymerase chain reaction; RFLP, Restriction fragment length polymorphism; DNA, Deoxy ribonucleic acid; NGS, Next-generation sequencing; WGS, Whole genome sequencing; POC, Point-of-care.

Recent advances in NGS have enabled massively parallel sequencing of the entire mitochondrial genome, facilitating highly sensitive detection of SNVs and accurate quantification of heteroplasmy levels (44). However, the clinical, ethical, and economic implications of non-actionable results require careful consideration.

High-resolution melt profiling is a rapid POCT that monitors thermal denaturation of DNA duplexes after PCR-based amplification (46). In the PALOH trial, this technique demonstrated a turnaround time of 23 min, facilitating pharmacogenetic prescription of AG in neonates (16). The findings suggest considerable potential for clinical implementation, although validation in larger, multicentre cohorts is required.

While advances in sampling methods and genotyping technologies have enhanced the accuracy and feasibility of m.1555A>G carrier detection, evidence remains limited regarding whether early identification and subsequent avoidance of aminoglycosides confer measurable benefits in long-term hearing outcomes.

### 4.4 Targeted screening for m.1555A>G

Pharmacogenetic testing is either “reactive”—performed after an adverse drug response occurs—or “pre-emptive”—done before prescribing to prevent negative outcomes from future treatments (11, 47). Both targeted and universal approaches to pre-emptive m.1555A>G screening have been proposed and evaluated; however, clinical implementation remains nascent.

In the targeted screening model, risk stratification of infants can be based on predefined clinical criteria, including NICU admission, a family history of NSHL, or a high likelihood of aminoglycoside exposure. A few isolated studies have explored this approach so far (16, 26, 33).

Prenatal NIPT-based screening may enable pharmacokinetically guided aminoglycoside prescribing, while allowing time for family counselling, preventive management, and cascade testing (47). POC genotyping brings a paradigm shift to the horizon, opening the avenue for pharmacogenetic prescribing in the acute neonatal care setting (16). This will be especially useful in extremely preterm infants who are at risk of multiple AG courses during NICU stay (1). While these pilot studies highlight the potential benefits of the targeted pre-emptive screening strategy, further validation studies are needed to establish its clinical utility and effectiveness to avert AIHL.

Advanced DNA microfluidics and fully integrated lab-on-a-chip platforms are actively being deployed for infectious disease detection, with several commercial and research developments pushing the boundaries of speed, sensitivity, and portability. The swift adoption of such technology simplified operational challenges and proved particularly valuable during the COVID-19 pandemic (15, 48). Likewise, POCT-guided AG prescribing has the potential to enhance neonatal care and may represent an important future direction for routine clinical practice.

### 4.5 Universal genetic screening for HL including MT-RNR1 mutations

In China, large-scale population-based studies paired universal auditory screening (OAE/AABR) with genetic screening for HL and reported broadly consistent conclusions (25, 27, 29–31, 34–36): Integrated screening programs increased the diagnostic potential for congenital HL by 2%–3%, reduced the time to establish etiological diagnosis, and helped in early identification of individuals with a genetic predisposition to preventable forms of HL. This screening strategy also facilitated tailored risk-mitigation measures, such as antibiotic alert cards and genetic counselling for MT-RNR1 carriers, and guidance to avoid head trauma in individuals with *SLC*26*A*4-associated enlarged vestibular aqueduct.

According to a systematic review by Pan et al. (*n* = 377,688), integrated NBHS detected an additional 0.31% of newborns with HL-associated genetic mutations that were missed by standard NBHS alone among Chinese ethnicities (49). Of these, 0.21% (95% CI: 0.18–0.26) were MT-RNR1 carriers, corresponding to one carrier per 500 newborns screened with the combined approach. The results cannot be extrapolated to other regions with low or unknown prevalence of MT RNR 1 mutations.

A critical appraisal of the large-scale screening programmes reported by Dai et al. (29) and Zhu et al. (31) yields several broadly applicable insights that may inform the implementation of m.1555A>G screening programmes across diverse healthcare systems.

1. Universal genetic screening generated a comprehensive genetic profile of the local population and furthered the understanding of its correlation with NBHS results, genotype-phenotype correlation, and association with haplogroups (Table 1). Leveraging archived DBS to establish population-based prevalence estimates of the m.1555A>G variant may provide a cost-effective and pragmatic evidence base to guide screening policy and clinical practice (50).
2. Integrated NBHS study by Wang et al. (25) conducted extended pedigree analysis of 16 index cases with m.1555A>G and identified 11 cases of deafness and 123 additional carriers with normal hearing. This highlights a potential benefit of pre-emptive universal screening for m.1555A>G. Cascade evaluation of relatives of identified carriers may enable timely risk mitigation and could contribute to reducing the public health burden of AIHL.
3. The programs were government-funded and integrated into existing public health infrastructure, with centralized genetic and audiological laboratory referral centers and trained manpower to provide follow-up, genetic, and audiological counselling. One large-scale screening programme reported a cost-benefit ratio of 1:7.27, indicating potential economic benefits from preventing hearing loss and its associated healthcare, educational, and societal costs (29). However, the applicability of these findings to other healthcare systems remains uncertain, underscoring the need for locally relevant economic evaluations.

### 4.6 Ethics of genetic screening in neonates

Genetic testing and screening in neonates demand a re-examination of the traditional paradigms, particularly regarding the adequacy of informed consent, the scope of parental authority as surrogate decision-makers, and the nature of risk itself. Decisions are debated based on the Wilson and Jungner principles and should be founded on the premise of best interests of the individual (17, 51, 52). According to the recommendations of the American Academy of Pediatrics (AAP) and the American College of Medical Genetics and Genomics (ACMG), predictive genetic screening in neonates is considered acceptable, only when it enables early interventions that avert substantial morbidity or mortality (52).

*MT-RNR*1 mutations (Online Mendelian Inheritance in Man, OMIM # 580000) follow an exclusive matrilineal pattern of inheritance, and m.1555A>G variants demonstrate significant clinical heterogeneity in expressivity, dose response spectrum, temporal variability, and severity of hearing impairment (10, 53). A positive result carries genetic, pharmacologic, audiologic, and reproductive implications, and therefore requires well-defined pre and post-test genetic counselling and structured longitudinal follow-up embedded into the healthcare system.

During the pre-test session, parents should understand the need for long-term audiological surveillance in carriers, regardless of the NBHS result. They should also be explained of potential pitfalls- e.g., the possibility of a false negative result if the tissue mutation load is heterogeneous or the heteroplasmy percentage is below analytic detection (47). The post-test counselling for positive results should include two essential components: cascade testing of maternal relatives and reproductive risk considerations for female m.1555A>G carriers. Moreover, robust data protection systems, continuity of care, and legal frameworks should be embedded in the platform to guarantee quality while upholding ethical and legal standards.

Integrated newborn hearing screening programmes incorporating genome-wide approaches such as NGS and WGS may further advance precision medicine and personalised healthcare in the neonatal period. However, their broader clinical, ethical, psychosocial, and societal implications remain incompletely understood and warrant careful evaluation beyond the scope of the present review (54).

## 5 Limitations

In synthesizing the current body of research on the m.1555A>G variant and its association with AIHL in neonates, this review acknowledges several key limitations. The evidence base is largely observational, with considerable heterogeneity in both study design and population. Furthermore, the generalizability of the findings is limited by the small number of m.1555A>G carriers in several studies and by the geographic concentration of the large population-based studies in China. Some population-based studies were feasibility studies, and data on audiological follow-up and economic-effectiveness remained preliminary. The possibility of overlapping study populations across publications may have further introduced duplication bias. Most importantly, neonatal studies are constrained to reporting NBHS outcomes, which are not predictive of AIHL. Long-term follow-up of neonatal cohorts is inherently challenging, and studies reported considerable attrition during follow-up (Table 1). Consequently, long-term hearing outcomes may have been incompletely captured, potentially affecting evidence regarding the clinical consequences of the *m.*1555*A>G* variant.

The evidence informing screening strategies is observational; to date, no study has directly compared universal and targeted screening approaches. Long-term attrition is particularly important because delayed-onset hearing loss is central to the rationale for screening; without longitudinal follow-up, the very outcomes that justify screening programs may go undetected, biasing estimates of benefit.

The literature search was restricted to peer-reviewed publications and did not include grey literature, dissertations, or publications in regional languages; relevant research may therefore have been overlooked.

## 6 Conclusions

Pathogenic *MT-RNR*1 variants, particularly m.1555A>G, confer susceptibility to AIHL and occurred at a study-level median frequency of approximately 0.2% in population-based newborn screening cohorts, with marked variation by ethnicity and geographic region; given the widespread use of aminoglycosides in preterm and critically ill neonates, conventional NBHS alone is insufficient to identify infants at risk.

\Molecular diagnostics, including targeted genotyping and universal newborn genetic screening, offer reliable methods for identifying mutation carriers and support preventive pharmacogenetic prescribing. While universal genetic screening provides the broadest coverage, risk-stratified testing may represent a pragmatic initial approach. However, the clinical utility and broader impact of both approaches remain to be established.

As genomic technologies become increasingly embedded in clinical care, pharmacogenetic-guided aminoglycoside prescribing represents a promising step towards precision medicine in neonatology. By identifying newborns genetically susceptible to AIHL, this approach may enable safer antibiotic use and reduce the burden of preventable hearing loss. Until such testing is routinely implemented, careful aminoglycoside prescribing with consideration of family history and other risk factors remains crucial.
