# Supplementary material for: Mitochondrial mutation m.1555A>G and aminoglycoside-induced hearing loss in neonates: an integrative review
Source: Front Pediatr. 2026 Oct 28;14:1973908. doi: 10.3389/fped.2026.1973908 (PMC13752603; doi:10.3389/fped.2026.1973908)
Supplement: Supplementary file 1 [file Table1.docx]

**Supplementary Material**

**Risk of bias assessment using JBI Critical Appraisal tools**

**Summary of the 18 included studies**

| **Study Design** | **Number of Studies** | **Risk of bias** |
| --- | --- | --- |
| Cohort studies | 12 | Moderate (Several N/U/NA) |
| Cross-sectional studies | 5 | Moderate (Several N/U/NA) |
| Case-control studies | 1 | Low (Mostly Y) |

**Supplementary Table S1 for Cohort Studies:**

| **Study** | **Q1** | **Q2** | **Q3** | **Q4** | **Q5** | **Q6** | **Q7** | **Q8** | **Q9** | **Q10** | **Q11** |
| --- | --- | --- | --- | --- | --- | --- | --- | --- | --- | --- | --- |
| Johnson et al., 2010 | Y | Y | Y | U | Y | Y | Y | N | U | N | Y |
| Nivoloni et al., 2010 | Y | Y | Y | NA | NA | N | Y | Y | Y | Y | Y |
| Wang et al., 2011 | Y | Y | Y | NA | NA | U | Y | Y | U | NA | Y |
| Göpel et al., 2014 | Y | Y | Y | Y | Y | Y | Y | Y | Y | Y | Y |
| Li et al., 2015 | Y | Y | Y | NA | NA | U | Y | Y | U | U | Y |
| Soini et al., 2017 | Y | Y | Y | N | N | U | Y | Y | Y | NA | Y |
| Dai et al., 2019 | Y | Y | Y | NA | NA | U | Y | Y | N | Y | Y |
| Zhu et al., 2021 | Y | Y | Y | NA | NA | Y | Y | Y | Y | Y | Y |
| McDermott et al., 2022 | Y | Y | Y | N | N | U | Y | Y | U | Y | Y |
| Kun et al., 2023 | Y | Y | Y | NA | NA | U | Y | N | N | Y | Y |
| Duan et al., 2025 | Y | Y | Y | U | U | Y | Y | N | N | Y | Y |
| Shi et al., 2025 | Y | Y | Y | U | Y | U | Y | Y | U | U | Y |

Y = Yes N = No U = Unclear NA = Not applicable

**Supplementary Table S2 for Cross-sectional studies:**

| **Study** | **Q1** | **Q2** | **Q3** | **Q4** | **Q5** | **Q6** | **Q7** | **Q8** |
| --- | --- | --- | --- | --- | --- | --- | --- | --- |
| Gravina et al., 2007 | Y | Y | Y | Y | N | N | Y | Y |
| Maniglia et al., 2008 | Y | Y | Y | Y | N | N | Y | Y |
| Ealy et al., 2011 | U | Y | Y | Y | U | N | Y | Y |
| Liu et al., 2019 | Y | Y | Y | Y | Y | Y | Y | Y |
| Huang et al., 2022 | Y | Y | Y | Y | Y | Y | Y | Y |

Y = Yes N = No U = Unclear NA = Not applicable

**Supplementary Table S3 for Case-Control Study:**

| Study | Q1 | Q2 | Q3 | Q4 | Q5 | Q6 | Q7 | Q8 | Q9 | Q10 | Score |
| --- | --- | --- | --- | --- | --- | --- | --- | --- | --- | --- | --- |
| Chant et al., 2022 | U | Y | Y | Y | Y | Y | Y | Y | N | Y | 8/10 |

Y = Yes N = No U = Unclear NA = Not applicable

**Reference:**

Moola S, Munn Z, Tufanaru C, Aromataris E, Sears K, Sfetcu R, et al. Chapter 7: Systematic reviews of etiology and risk. In: Aromataris E, Munn Z, editors. *JBI Manual for Evidence Synthesis*. Adelaide (AU): JBI; 2020. Available from: <https://doi.org/10.46658/JBIMES-20-08>
